# Macrophage Activation in the Synovium of Healthy and Osteoarthritic Equine Joints

**DOI:** 10.3389/fvets.2020.568756

**Published:** 2020-11-26

**Authors:** Bruno C. Menarim, Kiersten H. Gillis, Andrea Oliver, Ying Ngo, Stephen R. Werre, Sarah H. Barrett, Dwayne H. Rodgerson, Linda A. Dahlgren

**Affiliations:** ^1^Department of Large Animal Clinical Sciences, Virginia-Maryland College of Veterinary Medicine, Virginia Tech, Blacksburg, VA, United States; ^2^Laboratory for Study Design and Statistical Analysis, Virginia-Maryland College of Veterinary Medicine, Virginia Tech, Blacksburg, VA, United States; ^3^Department of Biomedical Sciences and Pathobiology, Virginia-Maryland College of Veterinary Medicine, Virginia Tech, Blacksburg, VA, United States; ^4^Hagyard Equine Medical Institute, Lexington, KY, United States

**Keywords:** joint homeostasis, osteoarthiritis, synovitis, inflammatioin, activation, polarization

## Abstract

Synovitis is a major component of osteoarthritis and is driven primarily by macrophages. Synovial macrophages are crucial for joint homeostasis (M2-like phenotype), but induce inflammation (M1-like) when regulatory functions become overwhelmed. Macrophage phenotypes in synovium from osteoarthritic and healthy joints are poorly characterized; however, comparative knowledge of their phenotypes during health and disease is paramount for developing targeted treatments. This study compared patterns of macrophage activation in healthy and osteoarthritic equine synovium and correlated histology with cytokine/chemokine profiles in synovial fluid. Synovial histology and immunohistochemistry for M1-like (CD86), M2-like (CD206, IL-10), and pan macrophage (CD14) markers were performed on biopsies from 29 healthy and 26 osteoarthritic equine joints. Synovial fluid cytokines (MCP-1, IL-10, PGE_2_, IL-1β, IL-6, TNF-α, IL-1ra) and growth factors (GM-CSF, SDF-1α+β, IGF-1, and FGF-2) were quantified. Macrophage phenotypes were not as clearly defined *in vivo* as they are *in vitro*. All macrophage markers were expressed with minimal differences between OA and normal joints. Expression for all markers increased proportionate to synovial inflammation, especially CD86. Synovial fluid MCP-1 was higher in osteoarthritic joints while SDF-1 and IL-10 were lower, and PGE_2_ concentrations did not differ between groups. Increased CD14/CD86/CD206/IL-10 expression was associated with synovial hyperplasia, consistent with macrophage recruitment and activation in response to injury. Lower synovial fluid IL-10 could suggest that homeostatic mechanisms from synovial macrophages became overwhelmed preventing inflammation resolution, resulting in chronic inflammation and OA. Further investigations into mechanisms of arthritis resolution are warranted. Developing pro-resolving therapies may provide superior results in the treatment of OA.

## Introduction

Osteoarthritis (OA) is a leading cause of lameness and morbidity and presents significant treatment challenges in horses and people (1, 2). The pathophysiology of OA is incompletely understood; however, there is increasing evidence that macrophages play a central role in the synovial inflammation leading to OA (3–6). Macrophage depletion in studies of rheumatoid (7) and experimental arthritis (8, 9) have established that macrophages drive synovial inflammation (7, 8, 10) by showing dramatically decreasing expression of OA biomarkers in the absence of macrophages (6, 11). Although other cells, such as chondrocytes, can further amplify the inflammatory reaction, they cannot induce it in the absence of macrophages (5, 6, 12–14). More recent studies have shown that activation of macrophages in osteoarthritic synovium is directly related to disease activity, severity, and pain (15, 16). Conversely, macrophages are also key regulators of joint homeostasis and chondrogenesis (4, 17). In healthy conditions, macrophages promote synovial integrity through phagocytic activity (i.e., clearance of foreign material, tissue debris, and efferocytosis), and secretion of synovial fluid, cytokines, chemokines, and growth factors (4, 17). When these homeostatic functions become overwhelmed, synovial macrophages upregulate inflammation, recruiting other immune cells to respond to the increased demands for repair and recovery of homeostasis (6, 18–20).

Upon defined stimulation *in vitro*, macrophages activate into a spectrum of phenotypes, with the extremes represented by cells displaying classical pro-inflammatory (M1) or pro-resolving/healing (M2) responses (21). *In vivo*, macrophages respond to oscillating environmental stimuli, displaying marked phenotype plasticity, and play such a fundamental role in resolving inflammation and promoting tissue repair, that macrophage exhaustion or depletion results in severely compromised wound healing or chronic inflammation (22–25). *Ex vivo* chondrogenesis of synovial progenitor cells is impeded by classically activated (M1-like) macrophages from the osteoarthritic synovium, while alternatively activated (M2-like) macrophages are required for efficient chondrogenesis (17, 26). Inflammation in arthritic joints is dampened by M2-like macrophages, improving clinical and histological signs of joint disease (19, 27, 28). Collectively, these findings suggest that enhancing the M2-like response in diseased joints may provide a mechanism for resolving joint inflammation and restoring a healthy synovial environment with improved capacity for tissue repair.

Specific information regarding macrophage phenotypes in joint disease is limited to *in vitro* studies, experimental animal models, or end stage OA in people (6, 10, 17, 26, 29). Comparisons of macrophage responses between diseased and healthy joints include extrapolations from other types of arthritides, such as rheumatoid arthritis. Additional reports are limited to low numbers of macrophages in the synovial fluid, shedding from the synovium following mechanical detachment or hyperactivation. Reports evaluating synovial fluid macrophages may not represent the response of the synovial membrane tissue itself (30, 31). Defining patterns of macrophage activation in the synovium of healthy and naturally developing osteoarthritic joints will enhance the understanding of the roles of macrophages *in vivo*, which is paramount for optimizing therapeutic strategies targeting macrophage-driven joint homeostasis (28).

The objective of this study was to compare the expression of macrophage markers in the synovium of healthy equine carpal and metacarpophalangeal (MCP) joints and those with naturally occurring OA. The well-defined equine model for the study of OA (1) used in this study will enable translation of information to the treatment of both equine and human patients suffering from OA. We hypothesized that synovial macrophages in osteoarthritic joints would exhibit increased ratios of M1:M2(-like) marker expression compared to healthy joints and that differences in gross pathology, histology, and concentrations of pro- and anti-inflammatory cytokines in synovial fluid would be associated with differences in M1:M2(-like) macrophage ratios in synovium. Antibodies targeting markers of M1 (CD86), M2 (CD 206 and IL-10), and all mature macrophages (CD14) were used to identify macrophage phenotypes in the synovium and a multiplex bead-based assay was used to determine concentrations of synovial fluid cytokines, chemokines, and growth factors.

## Materials and Methods

### Experimental Design

Synovial fluid and synovial membrane biopsies were collected from 26 osteoarthritic joints (16 MCP joints and 10 radiocarpal/middle carpal joints) of horses undergoing arthroscopy or following euthanasia at the Hagyard Equine Medical Institute (Lexington, KY) or the Virginia-Maryland College of Veterinary Medicine (Blacksburg, VA). Written informed consent was received from owners prior to inclusion of horses in the study. Control samples from healthy joints (15 MCP and 14 carpal joints) were collected at the same hospitals from horses without history and evidence of lameness referable to the harvested joints and with grossly healthy articular surfaces at euthanasia. All procedures were performed under IACUC approval. Both healthy and OA samples were harvested from 13 horses, OA samples only from 8, and healthy samples only from 7 horses. The mean age of control horses used to harvest healthy samples (7.4 years) was similar to those with OA (6 years), and comparable to horses used for both purposes (8 years). Synovial inflammation was assessed by gross pathology, synovial membrane histology, and synovial fluid cytology and immunoassay quantification of concentrations of pro- and anti-inflammatory cytokines and growth factors. Synovial macrophage phenotype activation *in situ* was defined by immunohistochemistry.

### Inclusion Criteria

A total of 29 horses (11 females and 18 castrated males), 3–15 years old (skeletally mature, but not aged) were recruited and lameness exams performed, including response to joint manipulation, joint flexion, gait analysis at the trot, and radiography. OA joints were from horses exhibiting Grade 1-3 (out of 5) lameness (32), localized to the selected joint. Diagnostic analgesia was performed at the discretion of the referring veterinarian, and therefore not in all horses. Inclusion was based on arthroscopic or post mortem findings of cartilage abnormalities according to the OARSI scale (0-3, for metacarpophalangeal joints; 0-4 for carpal joints) (33). Only moderate OA joints (OARSI grade 2) were included, as representative of those most commonly treated clinically and when synovium cellularity is highest (34). As per the OARSI guidelines, carpal joints were selected according to degree of macroscopic cartilage erosion (grade 2= partial thickness), and MCP joints were included if presenting a score of 2 for one of the three macroscopic diagnostic parameters: wear lines (3–5 partial-thickness or 1–2 full-thickness wear lines), erosion (partial-thickness erosion, >5 mm in diameter), or palmar arthroses (partial-thickness erosion, purple discoloration, >5 mm in diameter). Horses with a history of septic arthritis, non-steroidal anti-inflammatory therapy, or intra-articular diagnostic anesthesia within 2 weeks, intra-articular corticosteroids within 2 months prior to sample collection, or evidence of osteochondrosis were excluded from the study. Only healthy horses with a body condition score between 4 and 6 (out of 9) were included.

### Sample Collection

Synovial fluid (2 mL) was aseptically collected and aliquoted (EDTA and Protein LoBind microfuge tubes, Eppendorf®, Westbury, CT). Anticoagulant-free synovial fluid was immediately centrifuged at 12,000 × g for 10 min at 4°C and the supernatant stored at −20°C for cytokine and growth factor quantification. Two synovial membrane biopsies were obtained from each OA joint adjacent to the major cartilage alterations (35), using 6 mm dermal biopsy punches. Two control samples were harvested at sites where each joint is traditionally most commonly affected (33). Samples were fixed (AZF Fixative® Newcomer Supply, WI) at room temperature for 24 h, rinsed, and stored in PBS at 4°C until processing.

### Synovial Fluid Analysis

Synovial fluid cytology was processed for total nucleated cell count (TNCC) by hemocytometer and total protein (TP) by refractometer. Differential cell counts were performed following Romanowski stain (Microscopy Hemacolor®, Merck, Germany). Concentrations of pro- (IL-1β, IL-6, GM-CSF, TNF-α) and anti-inflammatory cytokines (IL-10, IL-1ra), chemokines (MCP-1, SDF-1), growth factors (IGF-1, FGF-2), and PGE_2_ in synovial fluid were quantified. Thawed samples (200 μL) were hyaluronidase-digested (10 μL of 100 IU hyaluronidase/mL acetate buffer; Worthington Biochemical Corporation, Lakewood, NJ) for 30 min at 37°C, centrifuged at 12,000 × g for 10 min at 4°C, and the supernatant recovered. Based on previous experience and interfering factors in cytokine detection in synovial fluid (36, 37), spike-and-recovery assays were performed for the PGE_2_ ELISA and 4 representative serially-diluted targets in the multiplex assay (IL-1β, IL-6, IL-10, TNF-α). Based on the results, a dilution of 1:2 was selected for PGE_2_ quantification and no dilution was deemed necessary for the multiplex assay.

PGE_2_ was quantified by ELISA (R&D Systems, Minneapolis, MN). Hyaluronidase-digested samples were solid-phase extracted (500 μL synovial fluid in 490 μL 100% ethanol and 10 μL glacial acetic acid incubated at 23°C for 5 min), centrifuged at 2,500 × g for 8 min at room temperature, and the supernatant collected. Remaining analytes were quantified by bead-based multiplex assay (MILLIPLEX MAP Equine Cytokine/Chemokine Multiplex Assay with manufacturer modification to include IGF-1, SDF-1, and IL-1ra; Luminex 200 plate reader Millipore Sigma, Burlington, MA).

### Synovial Membrane Histology and Immunohistochemistry

Fixed synovial membrane biopsies were paraffin-embedded, sectioned at 5 μm, and H&E-stained. Synovitis was scored based on the OARSI histopathology guide and included cell infiltration, vascularity, hyperplasia, edema, and fibrosis (33). Immunostaining for macrophage markers was assessed using a previously described semi-quantitative approach considering cell staining intensity, cell compartment distribution of the staining, distribution of the staining pattern over the synovial villi, and tissue compartment distribution of the staining (28, 38). Cell staining intensity was scored as: absent (0); mild (1); moderate (2); or intense (3). Staining distribution across synovial villi was scored as restricted to the base of the synovial villus (1); reaching portions of the synovial villus tip (2); or throughout the entire synovial villus (3). Cell compartment distribution was scored as cytoplasm (1), nucleus (2), or both (3). Tissue compartment distribution was scored as restricted to the cell (1), more evident in the matrix (2), or evident on both cell and matrix. Staining patterns were scored on 3 different tissue sections and averaged. Composite scores for each marker were compared between normal and OA samples for both histology and immunohistochemistry. For immunohistochemistry, all tissue sections were baked at 66°C overnight, deparaffinized, and incubated in antigen recovery solution (Antigen Retrieval Citra Plus, BioGenex, Fremont, CA) at 95°C for 10 min. Slides were stained (Super Sensitive™ Polymer-HRP IHC Detection System, BioGenex) using antibodies targeting the following markers: pan macrophage (equine CD14, Wagner Lab, Cornell University); M1 (mouse anti-human CD86 [clone 2331(FUN-1), BD Biosciences, San Jose, CA]); M2 (mouse anti-human CD 206 [clone ab64693, Abcam, Cambridge, UK]); and IL-10 (mouse anti-equine IL-10, Wagner Lab). Markers were selected based on extensive literature review [CD14 (39–43), CD 86 (44–47), CD206 (48–53), and IL-10 (54–57)] and specificity or validated cross-reactivity to equine samples (58–61). Positive controls using tissues know to express each marker, and negative controls using tissues known to be negative for each marker and those stained without primary antibody were included. All samples were blindly scored (BCM), and scores corroborated by a experienced investigators and a board certified pathologist.

### Statistical Analysis

Data analysis was performed using SAS version 9.4 (SAS Institute, Inc, Cary, NC). Effects of different joints sampled (carpi vs. MCP) and effects of disease (healthy vs. OA) on outcomes were assessed using linear General Estimating Equations (GEE) in an incomplete block design. Each of the linear models specified joint, disease, and the interaction between joint and disease as fixed effects. Correlation between observations within horse (the blocking factor) were modeled by specifying a compound symmetry covariance matrix. The interaction between joint and disease was further analyzed (sliced) to extract comparisons between disease conditions within joint. Scatterplots and analysis of covariance models were used to determine associations between synovial fluid cytology, synovial membrane histology, and synovial membrane immunohistochemistry parameters with joint condition (healthy vs. OA). For the analysis of covariance models, immunohistochemistry parameters (macrophage markers) were specified as covariates (one parameter at a time) while disease was the design effect. Statistical significance was set to *p* < 0.05.

## Results

### Synovial Fluid Cytology

To determine whether synovial fluid macrophage counts differed between healthy and OA joints, standard synovial fluid cytology analysis was performed (Table 1). Overall, TP was overall significantly higher in OA compared to normal joints (*P* = 0.0331). TNCC was also higher in OA compared to normal joints, but failed to reach significance (*p* = 0.0532). No overall differences were detected between normal and OA joints for differential cell counts (relative counts of synovial fluid macrophages, lymphocytes and neutrophils), and macrophages were the predominant cell type in all groups.

**Table 1:** Synovial fluid cytology from healthy and OA equine metacarpophalangeal and carpal joints (median, 95% Confidence Interval).

**Synovial fluid cytology**						
		**Total protein g/dL**	**TNCC cells/μL**	**Macrophages%**	**Lymphocytes%**	**Neutrophils%**
Metacarpophalangeal joints	**Control**	2.1 (1.5–2.4)	91 (24–256)	65 (55–73)	28 (24–43)	0 (0–3)
	**OA**	2.7 (1.1–3.9)	68 (21–607)	68 (49–79)	27 (4–44)	0 (0–3)
	*P–value*	*P = 0.1402*	*P = 0.6253*	*P = 0.6278*	*P = 0.5702*	*P = 0.5805*
Carpal joints	**Control**	2.4 (1.6–2.8)	24 (19–221)	58 (50–67)	33 (28–48)	2 (0–3)
	**OA**	3.1 (1.7–3.8)	124 (14–204)	61 (46–77)	31 (16–39)	2 (0–19)
	*P–value*	*P = 0.0595*	*P = 0.3370*	*P = 0.8715*	*P = 0.2167*	*P = 0.2251*
Overall	**Control**	2.1 (1.9–2.4)	91 (24–156)	64 (55–71)	30 (25–43)	1 (0–3)
	**OA**	2.7 (1.8–3.6)	110 (36–173)	65 (54–73)	29 (18–39)	0 (0–3)
	*P–value*	***P=0.0331***	*P = 0.0532*	*P = 0.8780*	*P = 0.2699*	*P = 0.1995*

No significant differences were detected between samples from control and OA joints.

TNCC, Total Nucleated Cell Count. P-values < 0.05 highlighted in bold.

### Cytokine/Chemokine and Growth Factor Quantification

To assess the secretory response of synovial lining macrophages, concentrations of pro- (IL-1β, IL-6, GM-CSF, TNF-α, PGE_2_) and anti-inflammatory cytokines (IL-10, IL-1ra), chemokines (MCP-1, SDF-1) and growth factors (IGF-1, FGF-2) were quantified in synovial fluid (Table 2). GM-CSF was below detectable limits (3.7 pg/mL) for all samples. Detection of MCP-1, SDF-1α+β, IL-10, and PGE_2_, was possible in the majority of samples. The remaining analytes (IL-1β, IL-6, TNF-α, IL-1ra, IGF-1, and FGF-2) were detected in only a minority of samples, precluding statistical analysis. Concentrations of PGE_2_ did not vary overall or when comparing OA to healthy MCP or carpal joints. Concentrations of synovial fluid IL-10 were overall lower in OA than healthy joints; however, only significantly lower in OA compared to healthy MCP joints (*p* = 0.0462). The concentrations of key chemokines for recruitment of circulating monocytes (MCP-1) and homing of myeloid progenitors (SDF-1) were altered in the osteoarthritic synovial fluid. Overall MCP-1 concentrations were significantly higher in OA than healthy joints (*p* = 0.0443). In contrast, SDF-1 concentrations were significantly lower in the overall comparison of OA to healthy joints (*p* = 0.0243) and within healthy and OA MCP joints (*p* = 0.0378).

**Table 2:** Cytokine, chemokine, and growth factor concentrations in synovial fluid of healthy and OA equine joints (median, 95% Confidence Interval).

**Analytes**											
		**FGF−2**	**IGF-1**	**IL-1β**	**IL-6**	**IL1-ra**	**MCP-1**	**SDF-1**	**IL-10**	**PGE**_**2**_	**TNF-α**
***Min. D.C***.		11.5 pg/mL	0.3 pg/mL	15.5 pg/mL	2.3 pg/mL	0.02 pg/mL	9 pg/mL	20.5 pg/mL	23.2 pg/mL	39 pg/mL	1.5 pg/mL
Metacarpo- phalangeal joints	**Control** *N* = 15	*N* = 444^*^ (23–137)	U	*N* = 4335^*^ (55–617)	*N =* 5 6^*^ (3–19)	U	*N =* 10 799 (128–1,508)	*N =* 14 241 (129–292)	*N =* 15 86 (55–97)	*N =* 14 69 (53–73)	*N =* 5 3.5^*^ (2–10)
	**OA** *N =* 16	*N=* 3 20^*^ (13–53)	*N =* 1 506^*^	*N =* 5 4 (28–4014)	*N =* 5 25 (3–65)	*N =* 1 3^*^	*N =* 13 773 (128–1,463)	*N =* 13 137 (89–208)	*N =* 11 68 (40–96)	*N =* 13 71 (53–75)	*N =* 3 5^*^ (3–24)
	*P–value*	–	–	–	–	–	*P = 0.0803*	***P=0.0378***	***P=0.0462***	*P = 0.7206*	–
Carpal joints	**Control** *N =* 14	*N =* 2 78^*^ (16–141)	*N =* 2 1917^*^ (196–3639)	*N =* 7 199 (21–5501)	*N =* 6 13 (3–89)	*N =* 4 6^*^ (1–219)	*N =* 14 786 (230–1,867)	*N =* 14334 (152–467)	*N =* 14 64 (41–98)	*N =* 14 67 (53–73)	*N =* 5 9^*^ (6–35)
	**OA** *N =* 10	*N =* 3 18^*^ (16–42)	*N =* 4 270^*^ (83–863)	*N =* 6 169 (18–695)	*N =* 5 11 (3–172)	*N =* 1 15^*^	*N =* 10 933 (260–2,526)	*N =* 10 267 (73–498)	*N =* 10 64 (57–108)	*N =* 10 73 (62–83)	*N =* 5 5 (3–41)
	*P–value*	–	–	–	–	–	*P = 0.1360*	*P = 0.1943*	*P = 0.7362*	*P = 0.3740*	–
Overall	**Control** *N =* 29	*N =* 6 44 (16–141)	*N =* 2 1,917 (196–3,639)	*N =* 11 283 (42–1,014)	*N =* 11 8 (3–22)	*N =* 4 6^*^ (1–219)	*N =* 24 799 (240–1,508)	*N =* 28 276 (188–320)	*N =* 29 80 (55–92)	*N =* 28 68 (58–72)	*N =* 10 6 (3–11)
	**OA** *N =* 26	*N =* 6 19 (12–53)	*N =* 5 407 (83–863)	*N =* 11 64 (18–4014)	*N =* 10 18 (4–40)	*N =* 2 9^*^ (3–14)	*N =* 23 880 (442–1,096)	*N =* 23 150 (109–278)	*N =* 21 66 (57–92)	*N =* 23 71 (64–75)	*N =* 8 5 (3–25)
	*P–value*	–	–	–	–	–	***P=0.0443***	***P=0.0243***	*P = 0.2052*	*P = 0.5159*	–

Min. D.C., Minimum detectable concentration; U, Undetectcted; –, p–values could not be determined due to small number of samples in which the analyte was detected;

*, *the actual confidence level is <95%. N in analyte columns, number of samples in which the analyte was detected. P-values < 0.05 highlighted in bold*.

### Synovial Membrane Histology

**Figure 1:**
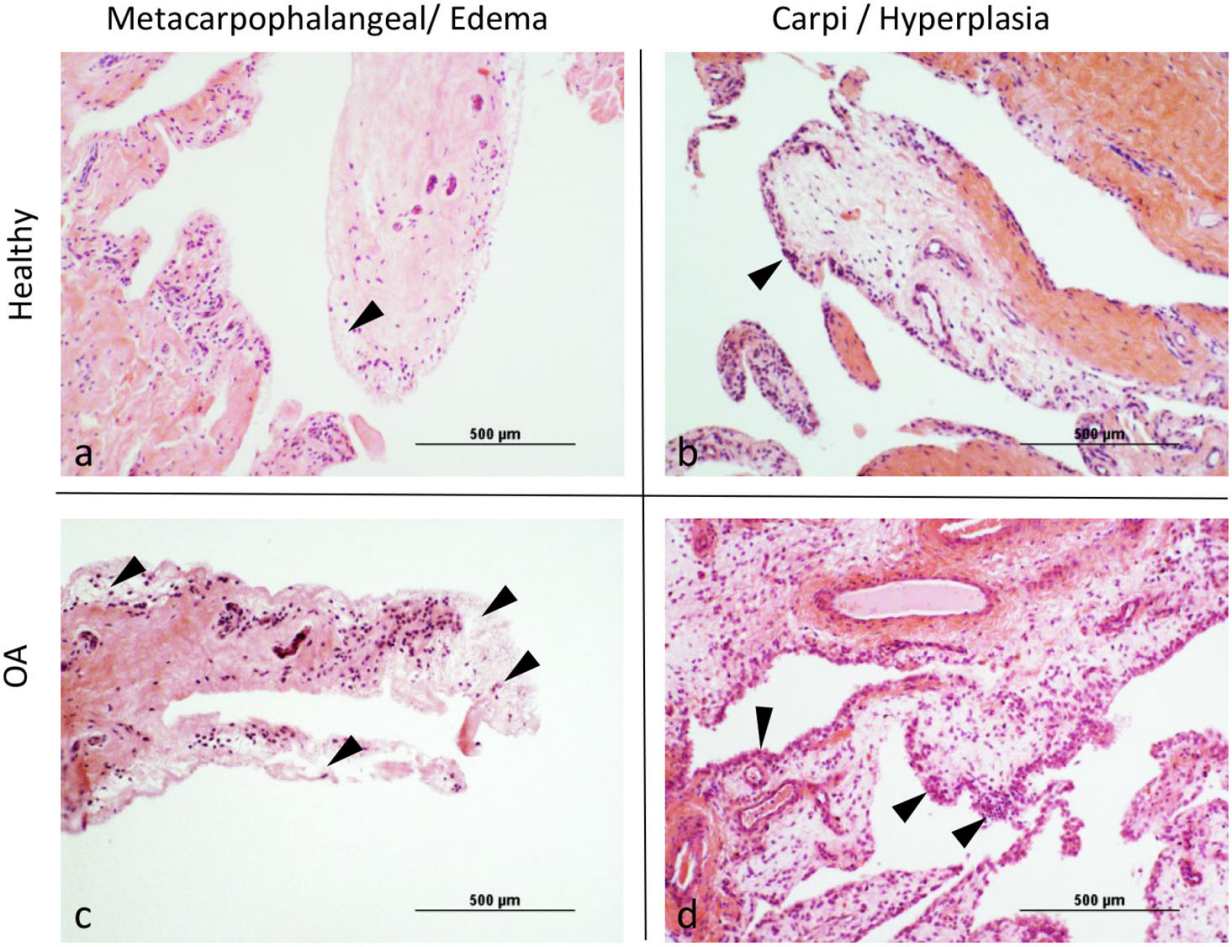
**(a–d)** Representative images demonstrating (arrowheads) the differences between healthy and osteoarthritic (OA) joints for Intimal Hyperplasia and Subintimal Edema.

**Figure 2:**
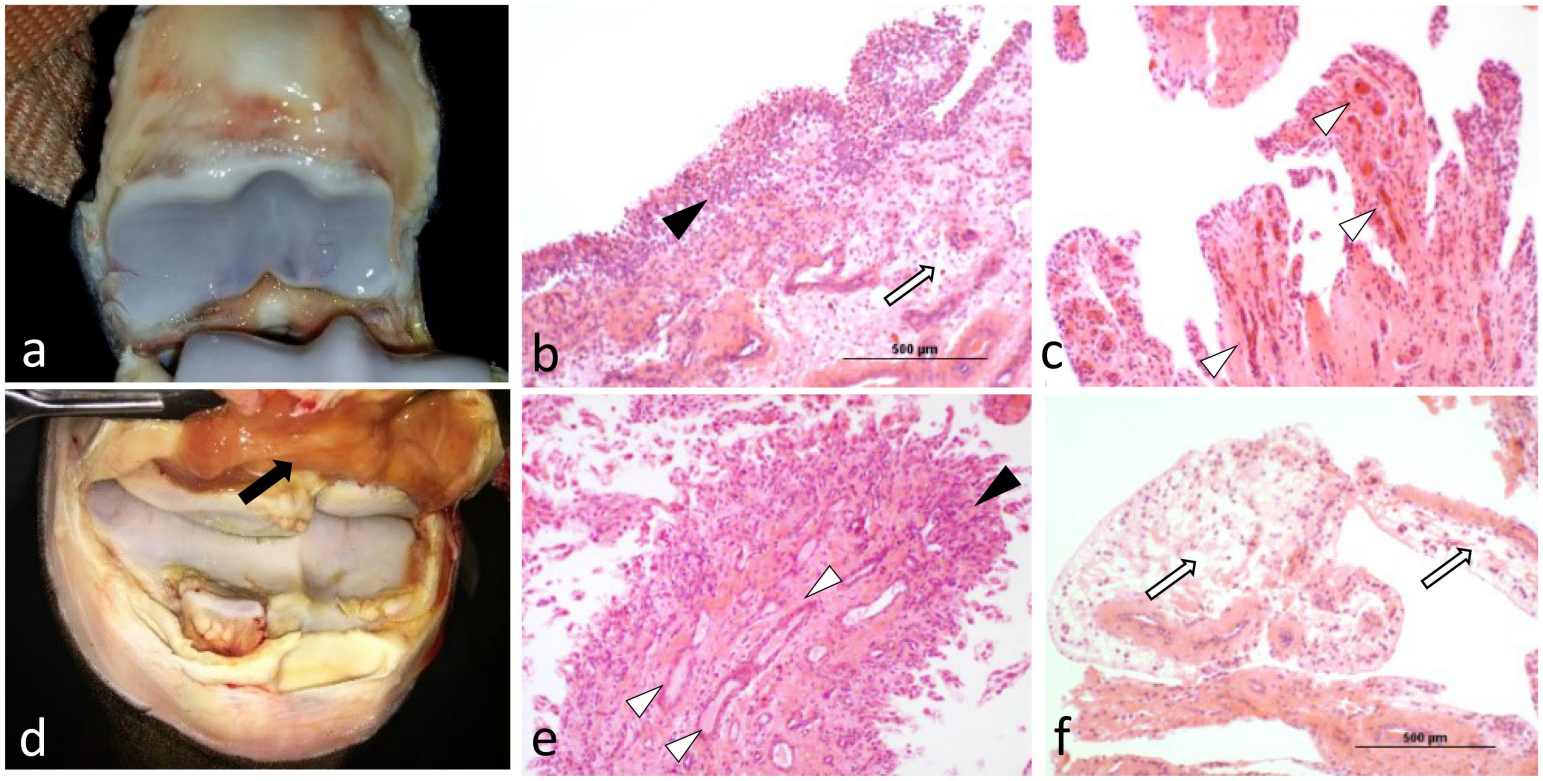
Compared to OA joints with no or minimal signs of gross inflammation **(a)**, OA joints exhibiting gross signs of synovitis (**d**; black arrow), exhibited increased histological changes such as severe cell infiltration and hyperplasia of the synovial intima with shedding of its outermost layer (**b**; black arrowhead), markedly increased vascularization (**c**; white arrowheads), or a combination of both **(e)**. Marked synovial and sub-synovial edema were also frequent findings (**b,f**; white arrows).

Overall scores for histological assessment of the synovium for intimal hyperplasia (*p* = 0.0076) were significantly higher in OA compared to normal joints (Table 3, Figure 1). Overall scores for subintimal edema (*p* = 0.0514), cell infiltration (*p* = 0.0818), vascularity (*p* = 0.1398), and fibrosis (*p* = 0.3053) were higher for OA joints, but were not significant (Table 3). The composite of these individual scores was significantly higher overall in OA compared to normal joints (*p* = 0.0122). Within MCP joints, only subintimal edema was significantly higher in OA joints compared to normal (*p* = 0.0158). Within carpal joints, intimal hyperplasia (*p* = 0.0103), and composite scores (*p* = 0.0420) were significantly higher in OA joints. In the subset of OA joints with gross signs of synovial inflammation (*n* = 8), there was a notable pattern of increased synovial vascularity and shedding of cells from the markedly hyperplastic outermost layer of the intima. In this outermost intimal layer, cell nuclei were often decondensed, with decreased hematoxylin uptake typical of hyperactivated cells (Figure 2) (62).

**Table 3:** Individual and composite histological parameters for H&E-stained equine synovial membrane (median, 95% Confidence Interval).

**Synovial membrane histology**							
		**Cell infiltration**	**Vascularity**	**Intimal hyperplasia**	**Subintimal edema**	**Fibrosis**	**Composite scores**
Metacarpo-phalangeal joints	**Control**	2 (1–2)	2 (1–3)	1 (0–1)	1 (0–2)	2 (2–3)	7 (4–11)
	**OA**	2 (1–3)	3 (1–4)	1 (0–3)	1.5 (1–3)	2 (1–3)	9.5 (6–14)
	*P–value*	*P = 0.3084*	*P = 0.1099*	*P = 0.1747*	***P=0.0158***	*P = 0.8501*	*P = 0.0711*
Carpal joints	**Control**	2 (1–2)	2 (1–2)	0.5 (0–1)	1 (1–2)	2 (1–3)	8.5 (5–9)
	**OA**	2 (1–3)	2 (0–3)	1 (1–2)	2 (0–3)	3 (1–3)	9.5 (7–12)
	*P–value*	*P = 0.1195*	*P = 0.7087*	***P=0.0103***	*P = 0.5231*	*P = 0.1973*	***P=0.0420***
Overall	**Control**	2 (1–2)	2 (1–2)	1^a^ (0–1)	1 (1–2)	2 (1–3)	8 (6–9)
	**OA**	2 (1–3)	2.5 (0–4)	1^a^ (0–2)	2 (1–3)	2.5 (2–3)	9.5 (7–12)
	*P–value*	*P = 0.0818*	*P = 0.1398*	***P=0.0076***	*P = 0.0514*	*P = 0.3053*	***P=0.0122***

Synovial Hyperplasia was significantly higher in OA carpi, while Subintimal Edema was significantly higher in OA metacarpophalangeal joints.

a *the categorical nature of the data produces median values that are equal between groups. P-values < 0.05 highlighted in bold*.

### Synovial Membrane Immunohistochemistry

**Figure 3:**
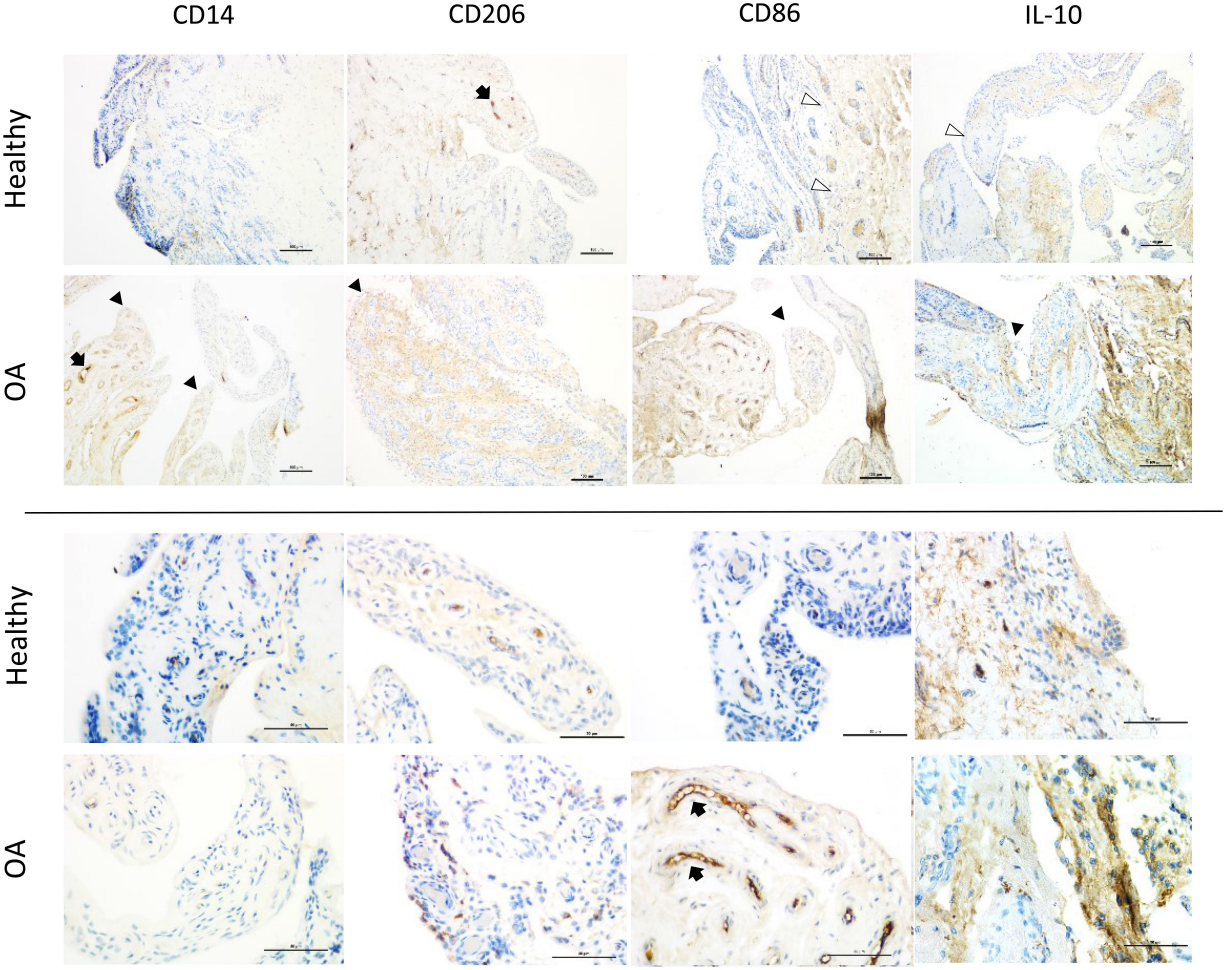
Representative immunohistochemistry sections from healthy and OA equine synovial membrane at low (top 2 rows; scale bar = 100 μm) and high magnification (bottom 2 rows; scale bar = 50 μm) from the same histological section and demonstrating the median staining scores for macrophage markers (CD14, CD86 [M1], CD206 and, IL-10 [M2]). Staining for all markers was most intense on the vascular endothelium (black arrows). In healthy joints, staining was largely limited to the base of synovial villi (white arrowheads), while in OA joints the tips of villi were also frequently stained (black arrowheads). In healthy joints, staining for macrophage markers at the tips of villi was subtle and primarily located at the synovial lining around cell nuclei. In contrast, staining in OA joints was more diffusely distributed in the synovial lining around cell nuclei.

**Figure 4:**
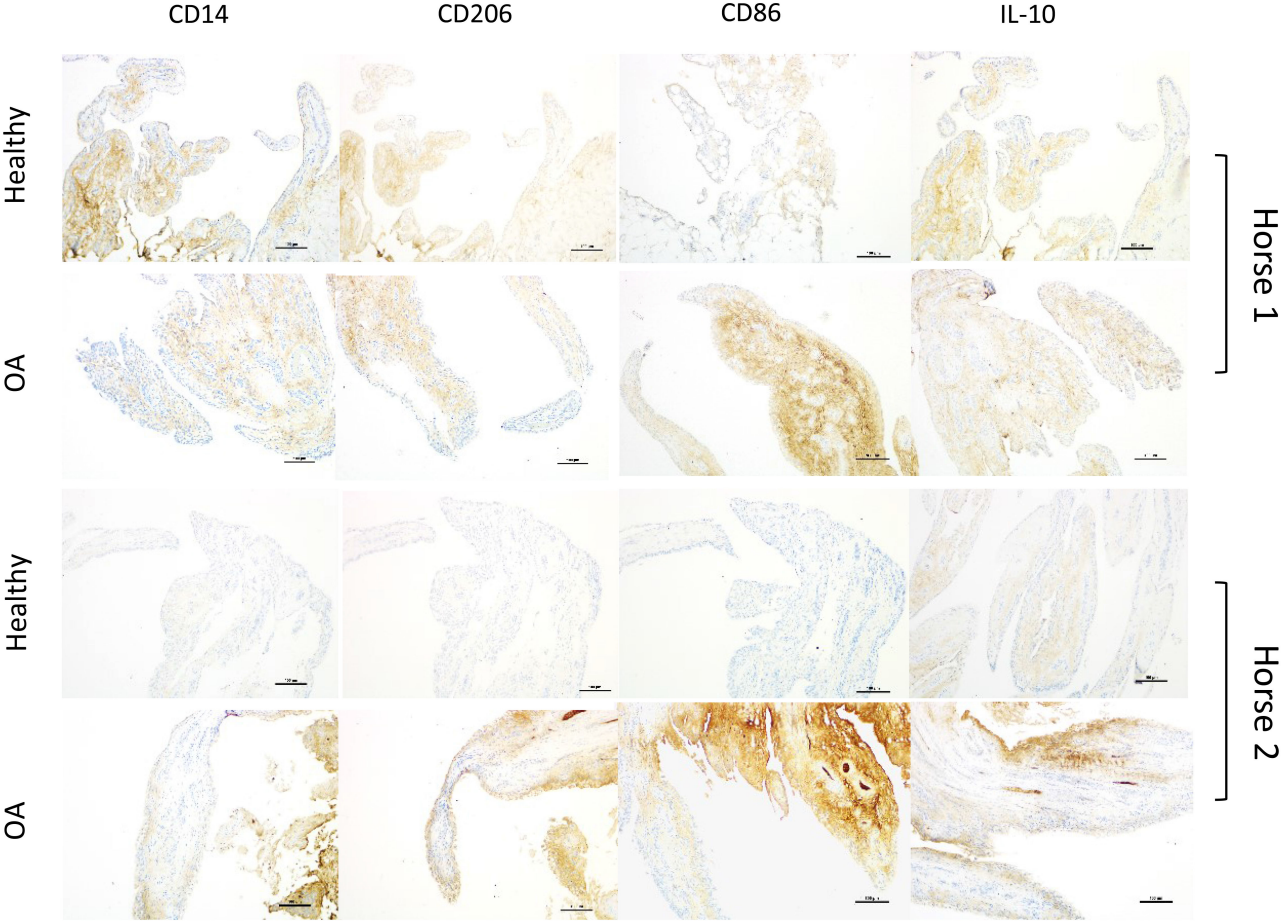
Sets of representative immunohistochemistry sections from healthy and grossly inflamed OA equine synovial membrane from the same horse (2 different horses; scale bar = 150 μm) demonstrating increased staining intensity and distribution for all selected markers in OA joints, denoting more consistently marked increases for CD86 staining.

The distribution of immunostaining for macrophage markers across the synovial lining differed between healthy and OA joints. In healthy joints, staining was largely limited to the base of synovial villi, while in OA joints the tips of villi were also frequently stained (**Figure 4**). When observed in healthy joints, staining for macrophage markers at the tips of villi was subtle and primarily located at scattered areas of the synovial lining around cell nuclei. In contrast, staining patterns in OA joints were more diffusely distributed in the synovial lining around cell nuclei. Overall, expression of CD14, CD86, and IL-10 were higher in OA compared to normal joints, yet only significantly for CD14 (*p* = 0.0157; Table 4). These differences were most apparent in the synovium from MCP joints, and again, only significant for CD14 when comparing healthy and OA MCP joints (*p* = 0.0279). Of note, in both MCP and carpal joints, expression of the M2 marker CD206 was higher in OA joints, although not significantly. Staining for all markers was most intense around blood vessels, especially over cells in the endothelium (Figure 3). Overall, CD14, CD86, and CD206 staining was limited primarily to the area immediately adjacent to cells and cell aggregates within the synovial intima and subintima, whereas staining for IL-10 was diffuse throughout the synovial tissue in both healthy and OA joints.

**Table 4:** Composite immunohistochemical scores of macrophage markers in healthy and OA synovial membrane (median, 95% Confidence Interval).

**Synovial membrane immunostaining**					
		**CD14**	**CD86**	**CD206**	**IL-10**
Metacarpo-phalangeal joints	**Control**	4 (0–5)	4 (0–6)	4 (4–6)	5 (4–6)
	**OA**	5 (0–7)	5 (0–7)	5 (0–6)	6 (4–6)
	*P–value*	***P=*** **0.0279**	*P =* 0.8593	*P =* 0.2987	*P =* 0.1551
Carpal joints	**Control**	5 (4–6)	6 (4–6)	4 (0–6)	6 (5–6)
	**OA**	5 (0–6)	6 (5–8)	5.5 (4–7)	6 (5–7)
	*P–value*	*P =* 0.1135	*P =* 0.2099	*P =* 0.1161	*P =* 0.8826
Overall	**Control**	5 (0–5)	5 (4–6)	5 (0–6)	5 (5–6)
	**OA**	6 (4–6)	6 (5–7)	5 (4–6)	6 (5–6)
	*P–value*	***P=*** **0.0157**	*P =* 0.3677	*P =* 0.5943	*P =* 0.3651

Osteoarthritic metacarpophalangeal joints exhibited increased expression of all markers, while only CD206 expression was higher in OA carpi. P-values < 0.05 highlighted in bold.

In the subset of OA joints with gross signs of synovitis, staining for CD86 was more markedly intense than remaining OA joints. A similar, but less consistent pattern was observed for CD14, IL-10, and CD206. For 4 horses, we were able to compare OA joints with gross signs of synovitis to the healthy contralateral joints of the same individual (Figure 4). Again, while increased expression of all markers in OA joints of these horses varied in intensity, CD86 expression was the most intense and consistently increased. Three of these four samples represented the highest CD86 staining scores among all samples of our study.

## Discussion

This is the first study in any species comparing macrophage phenotypes in the synovium from healthy joints to those with naturally occurring OA. Markers widely used to define M1- (CD86) and M2-like (CD206 and IL-10) macrophages were similarly expressed in both groups. Expression for all markers varied with degree of synovial inflammation. While their expression was mildly increased in OA joints with low-grade inflammation (majority), it was markedly increased in grossly inflamed OA joints, with CD86 most highly expressed. *In situ*, similar expression of M1- and M2-like macrophage markers (10, 26, 63) and their increased expression proportionate to inflammatory activation (6, 28, 64) are reported. Current knowledge suggests that, *in vivo*, macrophages are by default homeostatic cells that, following injury, drive inflammation with the purpose of counteracting tissue aggressors and further guide inflammation resolution (20, 65–70). Although no parameters revealed statistical associations with joint condition (healthy or OA), immunohistochemical and histologic findings were consistent with higher synovial fluid concentrations of MCP-1 and lower concentrations of SDF-1 in OA joints and lower IL-10 in OA metacarpophalangeal joints.

Under sustained inflammatory conditions, macrophages have lower expression of pro-resolving molecules such as IL-10 (64). This apparently impaired production by individual cells can be compensated for by increasing the overall numbers of macrophages to achieve inflammation resolution (28, 64). In the chronic inflammation of OA, higher CD14/CD186/CD206/IL10 expression (macrophage activation) and synovial intimal hyperplasia (macrophage recruitment), as observed in our study, is consistent with macrophages forming an isolating barrier at the site of the inflammatory response as shown by the black arrowheads in Figure 2b and as previously reported (20, 70). However, combined with decreased synovial fluid concentrations of IL-10, these observations may suggest that regulatory functions of these macrophages could be overwhelmed preventing recovery of joint homeostasis (22, 23, 28, 65–68, 71). Therefore, to efficiently achieve synovial inflammation resolution, recruitment of macrophages to the synovial environment likely needs to be higher than that resulting in intimal hyperplasia. Lower SDF-1 concentrations could be affecting efficient macrophage recruitment.

The concept of macrophage activation as either inflammatory (M1) or regulatory (M2) originated from monocyte-derived macrophages treated *in vitro* with defined and overwhelming cytokine stimuli (21, 52, 72). Clear identification of macrophage phenotypes *in vivo* is significantly more complex than proposed by *in vitro* models (10, 16, 22, 29). Increased CD14 expression during OA, combined with increased macrophage recruitment and activation corroborated by histology, as observed in our study, is consistent with previous reports of increased soluble CD14 in the synovial fluid of OA joints correlated with disease activity and clinical signs (73). Although CD86 and CD206 expression have historically been considered markers of M1- and M2-like macrophages (6, 26, 52, 72), this is an oversimplification of events that occur *in vivo* (16, 22, 74). CD86 is constitutively expressed by early myeloid cells and resting macrophages, including those in the synovial membrane and fluid of normal joints (28). Increased CD86 expression is part of the cellular checkpoints required for monocytic lineage commitment, activation, and inflammation resolution (22, 66). Therefore, as observed in the control joints in this study, and additional reports (22, 28, 64), isolated association of CD86 with an inflammatory phenotype or detrimental effects *in vivo* is likely misleading. Given the key role of CD86 in recruitment, activation, and survival of myeloid monocytes during both inflammation and its resolution, intense CD86 expression during acute (gross) inflammation suggests increased macrophage recruitment.

Similar to CD86, the mannose receptor (CD206) has a pivotal function in host defenses during inflammation, clearance of debris, wound healing and remodeling, and resolution of inflammation. CD206 is also constitutively expressed in mature mononuclear phagocytes and the intensity of its expression is proportionate to demands for anabolic cytokine secretion, efferocytosis, and sensing of damage-associated molecular patterns (68). Thus, the expression of both CD86 and CD206 increase with inflammatory stimuli, as a result of increased macrophage recruitment and response to injury (10), and therefore should be carefully analyzed over time in conjunction with clinical and analytical indicators of health and disease. Although expression of CD86 and CD206 was reported to associate to M1- and M2-like macrophages in the synovial fluid from healthy and OA joints (30), this observation is in disagreement with the profiles of macrophages in the synovium in this and other experimental studies (10, 26, 28, 64).

Like CD86 and CD206, expression of IL-10 in the synovial membrane in our study was directly associated with the degree of synovial inflammation. After injury, macrophage activation leads to increased expression of IL-1, IL-6, and TNF-α, which is followed by proportional increases in expression of IL-10 as a compensatory, negative feedback (75–77). Consequently, the production of these pro-inflammatory cytokines decreases (75). However, if the injurious challenge persists, this cytokine feedback loop is sustained, and may explain the increased synovium expression of IL-10 in our OA joints compared to healthy joints, especially those grossly inflamed (12, 28, 64, 76). Therefore, marked staining in grossly inflamed joints could suggest that the dynamics of cell recruitment and activation during inflammation (increased CD14, CD206, and CD86), and compensatory negative feedback (IL-10) are being persistently triggered in the vicious cycle of inflammation seen in OA (10, 12, 16, 66, 67, 75, 76, 78, 79). Considering the functions of these markers, their combined higher expression during OA suggests higher macrophage activation and not necessarily a phenotype as traditionally described *in vitro*.

The lower synovial fluid concentrations of IL-10 in OA metacarpophalangeal joints suggests that mechanisms compensating for tissue damage may be impaired or overwhelmed in OA joints. An *in vitro* study challenging monocytes from osteoarthritic and healthy human joints reported that patients with no significant IL-10 increase following challenge were three times more likely to develop OA compared to those responding with a significant increase (77). As a matter of fact, injection of arthritic joints with autologous bone marrow-derived macrophages results in marked clinical improvement, decreased markers of inflammation, and increased synovial fluid concentrations of IL-10 and IL-10^+^ macrophages (28, 80, 81). Inflamed equine joints treated with IL-10-expressing macrophages were comparable to healthy joints histologically, whereas saline-treated controls remained severely inflamed (28). Combined, these studies reinforce the important role of IL-10-producing macrophages in driving resolution of inflammation and promoting joint homeostasis (28, 80, 81).

In response to injury, resident synovial macrophages form a protective immunological barrier in the synovial lining, similar to the hyperplastic synovium, secluding intra-articular structures. Exchange of solutes and cells from the sub-synovial to intra-articular space is restricted and could explain higher IL-10 staining in the synovium from OA joints with lower synovial fluid IL-10 concentrations than healthy joints (20). During overwhelming inflammation, this tight-junction barrier is lost, allowing free exchange of cellular and molecular components between intra-articular and sub-synovial spaces (70). Importantly, each of these mechanisms can be affected by the stage of the inflammatory response (acute-chronic/mild-severe), which was not accounted for in our study design.

Increased overall synovial fluid MCP-1 concentrations in OA, concomitant with clinical signs of joint inflammation, is consistent with the literature (82–84). During synovial inflammation, MCP-1 contributes to recruitment and accumulation of circulating monocytes in the synovial membrane (76, 85). Although it has been suggested that MCP-1 has an important role in vicious cycles of inflammation (20, 86), this response is considered to be a homeostatic response to joint damage. As such, MCP-1-deficient mice are unable to home macrophages to sites of injury and are prone to impaired healing, infection, and chronic inflammation (76, 87, 88). Therefore, in the face of decreased SDF-1 and IL-10, increased concentrations of MCP-1 may be a compensating mechanism for recruitment of myeloid-derived macrophages to the injured joint (89, 90). Lower SDF-1 concentrations in synovial fluid from OA vs. healthy joints in our study is inconsistent with previous studies. SDF-1 has multifaceted roles in synovial tissue biology, including homeostatic and pro-inflammatory functions (91, 92). SDF-1 is reportedly expressed proportionate to disease activity, with higher concentrations in inflamed joints (91–93). Our results showing lower synovial fluid SDF-1 concentrations in OA joints is comparable to two other studies from our lab, where inflammation decreased synovial fluid SDF-1 (28, 64). SDF-1 is known to substantially improve tissue repair and plays a major role in recruiting and homing of myeloid cells involved in tissue repair and inflammation resolution (94), such as IL-10-producing myeloid-derived suppressor cells, critical for resolution of joint inflammation (81). The unbalanced production of all three substances can be related, and disturbances in their concentrations can reflect impaired macrophage recruitment or function. Further studies exploring the relationship of these findings are warranted.

Traditionally, IL-1β and TNF-α have been considered the main drivers of disease processes in OA (95–98). However, these two classic inflammatory cytokines were detected in less than half of our samples with no significant differences between healthy and OA samples, similar to previous reports (99, 100). Limitations in the detection of IL-1 and other cytokines in synovial fluid are widely reported, even in samples from patients experiencing marked inflammation (36, 37, 101, 102). Recent proteomic analysis of synovial fluid and genome-wide transcriptomic analysis of cartilage comparing samples from OA and healthy joints did not identify IL-1 or TNF-α as central targets (103, 104). PGE_2_ has also been used as an important marker of joint inflammation (11, 105, 106). However, PGE_2_ also plays anti-inflammatory and anabolic roles, such as inhibition of inflammatory cytokines and neutrophil infiltration to the site of injury, chondrocyte protection, and activation of pro-resolving macrophages (23, 52, 107, 108). PGE_2_ generated during the early inflammatory response can induce inflammation resolution by upregulating the synthesis of potent mediators of resolution (23). Therefore, PGE_2_ is involved in both inciting and resolving inflammation, and concentrations in synovial fluid vary with the stage of response to injury, and may explain the lack of differences between healthy and OA joints in our study.

Although differences between normal and OA joints were observed for both carpal and metacarpophalangeal joints, differences were more often identified in metacarpophalangeal joints. One potential reason for this observation is that, due to a more distal location and higher range of motion, metacarpophalangeal joints are more exposed to higher mechanical loads and stress, and thus the response to trauma and tissue microdamage may be more marked. In fact, metacarpophalangeal joints are the most commonly affected site of injury in many equine disciplines (109–111).

Even though our experimental design was aimed at minimizing variability, synovial histological parameters can vary with joint and site within the joint, and could have contributed to a degree of variability among samples, preventing statistical inference. While assessing the expression of macrophage markers in synovial fluid cells using flow cytometry would have contributed to our findings in the synovial membrane, recent reports are in agreement with the pattern of expression identified in our study (28, 112). Our study was not designed to infer causality of our findings in the development and progression of OA, and therefore the meaning of our observations is interpreted based on the literature and additional studies from our lab. Quantifying soluble CD14 in the synovial fluid could have reinforced the role of macrophage activation in joint inflammation and disease progression, yet such observations have already been reported, and similar to our study, were associated with increased MCP-1 concentrations in osteoarthritic synovial fluid (84). Immunoblots comparing the activity of the TLR-4 – NFκB-IL-10 axis between the synovium of healthy and OA joints, as well as quantification of other pro-resolving mediators in the samples of this study, would have provided additional information for understanding the mechanism by which drivers of joint homeostasis become overwhelmed. Futures studies comparing synovium single cell transcriptome analysis and synovial fluid lipid profiling between normal and OA joints will further define the role of synovial macrophages in joint disease.

## Conclusion

Combined with previously reported studies, our results suggest that synovial macrophages are strictly neither M1 nor M2, but represent a hybrid state of activation that overall displays a regulatory response and that ultimately targets resolution of the inflammatory process (22, 28, 64, 70, 113). The majority of parameters investigated in our study, pragmatically called pro- or anti-inflammatory, are building blocks of a complex immune response and must be carefully interpreted, with attention to the phases of inflammation, including its resolution. Secretion of pro- and anti-inflammatory/pro-resolving mediators increase proportionally, and almost simultaneously after macrophage activation in response to injury, decreasing to baseline after resolution (23, 67, 78). In OA joints, increased synovial fluid MCP-1 associated with synovial intimal hyperplasia suggests recruitment of macrophages to the synovium in response to injury. Nonetheless, decreased concentrations of pro-resolving mediators such as IL-10 and SDF-1 implies that pro-resolving mechanisms compensating for tissue damage leading to resolution may be impaired or overwhelmed. Furthermore, inflammation resolution is an active process, largely orchestrated by macrophages, and requires lipid mediators produced during the acute inflammatory response. Thus, the idea of inhibiting inflammation as a therapy may need to be revisited (23). An alternative way of thinking about the treatment of OA is to stimulate endogenous resolution of inflammation by increasing the innate homeostatic mechanisms of the joint, rather than simply blocking inflammation through the use of non-steroidal anti-inflammatory drugs and corticosteroids. Developing approaches to maximize the homeostatic response by healthy macrophages in OA joints has the potential to resolve joint inflammation and re-establish an anabolic synovial environment and overall joint health.

## Data Availability Statement

The raw data supporting the conclusions of this article will be made available by the authors, without undue reservation.

## Ethics Statement

The animal study was reviewed and approved by Virginia Tech Institutional Animal Care and Use Committee. Written informed consent was obtained from the owners for the participation of their animals in this study.

## Author Contributions

BM, DR, SW, and LD contributed substantially to study conception and design. BM and DR collected samples. BM was primarily responsible for data acquisition, analysis, and interpretation. KG, AO, and YN assisted BM with data collection and assembly. SB supervised the synovial fluid cytology performed by BM and KG. SW performed statistical analysis and consulted on its interpretation. BM and LD were responsible for manuscript preparation. All authors reviewed the final manuscript. All authors contributed to the article and approved the submitted version.

## Conflict of Interest

The authors declare that the research was conducted in the absence of any commercial or financial relationships that could be construed as a potential conflict of interest.

## Abbreviations

MCP: metacarpophalangeal joints
M1: classically activated/pro-inflammatory
M2: suppressive/healing
MCP-1: macrophage chemoattractant protein 1
OA: osteoarthritis/osteoarthritic
PGE_2_: prostaglandin E_2_
SDF-1: stromal cell–derived factor 1
TNCC: total nucleated cell count
TP: total protein
CD14: LPS co-receptor along with Toll-like receptor 4— proposed mature macrophage marker
CD86: /T cell costimulatory receptor (B7.2)—proposed M1 marker
CD206: Mannose receptor 1 (MRC1)—proposed M2 marker.
